# Cationic Surfactant-Driven Evolution of NiFe_2_O_4_ Nanosheets for High-Performance Asymmetric Supercapacitors

**DOI:** 10.3390/ma18091987

**Published:** 2025-04-27

**Authors:** Pritam J. Morankar, Rutuja U. Amate, Aviraj M. Teli, Mrunal K. Bhosale, Sonali A. Beknalkar, Chan-Wook Jeon

**Affiliations:** 1 School of Chemical Engineering, Yeungnam University, 280 Daehak-ro, Gyeongsan 712-749, Republic of Korea; pritam.nanoworld@gmail.com (P.J.M.); rutu.nanoworld@gmail.com (R.U.A.); mrunal.snst.1@gmail.com (M.K.B.); 2 Division of Electronics and Electrical Engineering, Dongguk University-Seoul Campus, Seoul 04620, Republic of Korea; avteli.teli@gmail.com (A.M.T.); sonalibeknalkar@gmail.com (S.A.B.)

**Keywords:** surfactant-assisted NiFe, hydrothermal, nanosheets, charge storage, asymmetric pouch-type supercapacitor

## Abstract

This work explores the role of cetyltrimethylammonium bromide (CTAB) as a morphology-directing agent in the hydrothermal synthesis of NiFe_2_O_4_ electrodes for high-performance supercapacitor applications. By fine-tuning CTAB concentrations (0.5%, 1%, and 1.5%), a tunable nanosheet morphology was achieved, with the NiFe-1 sample exhibiting uniformly interconnected nanosheets that enhanced ion diffusion, charge transport, and surface redox activity. Structural and surface analyses confirmed the formation of single-phase cubic NiFe_2_O_4_ and the presence of Ni^2+^ and Fe^3+^ oxidation states. Electrochemical characterization in a 2 M KOH electrolyte revealed that the NiFe-1 electrode achieved an areal capacitance of 8.21 F/cm^2^ at 20 mA/cm^2^, with an energy density of 0.34 mWh/cm^2^ and a power density of 5.5 mW/cm^2^. The electrode retained 79.61% of its capacitance after 10,000 cycles, demonstrating excellent stability. An asymmetric pouch-type supercapacitor device (APSD), assembled using NiFe-1 and activated carbon, exhibited an areal capacitance of 1.215 F/cm^2^ and delivered an energy density of 0.285 mWh/cm^2^ at a power density of 6.5 mW/cm^2^ across a wide 0–1.8 V voltage window. These results confirm that CTAB-assisted nanostructuring significantly improves the electrochemical performance of NiFe_2_O_4_ electrodes, offering a scalable and effective approach for next-generation energy storage applications.

## 1. Introduction

The growing demand for efficient energy storage technologies has driven intense research into advanced materials for high-performance supercapacitors. Among various options, supercapacitors are particularly attractive due to their excellent power density, rapid charge–discharge behavior, and long operational lifespan. However, their relatively low energy density remains a critical limitation, prompting the need for innovative electrode materials with enhanced electrochemical characteristics [1,2,3,4]. Transition metal oxides and spinel ferrites have emerged as promising candidates for electrode materials in supercapacitors owing to their multiple redox-active sites, stable structural frameworks, and tunable electronic properties that facilitate efficient charge storage [5,6,7]. In particular, nickel ferrite (NiFe_2_O_4_), a spinel-structured transition metal oxide, has attracted significant attention due to its high theoretical capacitance, intrinsic magnetic behavior, and structural robustness. The synergistic interaction between Ni^2+^ and Fe^3+^ ions in the spinel lattice supports fast and reversible faradaic redox reactions, making it highly suitable for pseudocapacitive applications [8,9].

To improve the practical performance of NiFe_2_O_4_-based electrodes, numerous strategies have been explored in the recent literature [10]. Zi-You et al. synthesized NiFe_2_O_4_ on carbon cloth via surfactant-assisted hydrothermal methods and achieved specific capacitances of 1135.5 F/g (1 M H_2_SO_4_) and 922.6 F/g (6 M KOH) at 2 mA/cm^2^, with over 80% capacitance retention [11]. Vidyadevi et al. prepared NiFe_2_O_4_ thin films using spray pyrolysis and reported a capacitance of 707 F/g at 2 mV/s (1 M KOH), with an energy density of 83.88 Wh/kg and a power density of 5.294 kW/kg [12]. Bandgar et al. examined the effect of morphology on performance by synthesizing NiFe_2_O_4_ in forms such as nanosheets, nanoflowers, and nanofeathers. The nanosheet morphology, with a surface area of 47 m^2^/g, achieved a capacitance of 1139 F/g in 6 M KOH, with 98% retention over 7000 cycles [13]. Similarly, Arun et al. explored ferric ion concentration effects during the chemical oxidation of NiFe_2_O_4_. Their optimized NF50 electrode exhibited a 277 F/g capacitance and 101% retention after 5000 cycles [14]. Gao et al. synthesized NiFe_2_O_4_ with controlled morphologies via hydrothermal precipitation and observed that nanosheet-like NiFe_2_O_4_ (NFO-U) offered a capacitance of 240.9 F/g at 1 A/g [15]. Despite these promising results, NiFe_2_O_4_-based electrodes still face performance limitations due to their relatively poor intrinsic conductivity and limited ion transport kinetics [16]. These drawbacks constrain their rate performance and long-term cycling behavior, motivating efforts to enhance electrochemical responses through nanostructure engineering, surface modification, and hybridization strategies [1,5,6]. Among these, controlling the synthesis route to yield the desired morphologies has proven to be an effective method to improve both ion diffusion and electron transfer [17].

One of the most practical and scalable approaches to morphology tuning involves the use of structure-directing agents, particularly surfactants. Surfactant-assisted synthesis enables precise control over crystal growth, size distribution, and surface functionality. Surfactants can mitigate particle agglomeration, enhance the active surface area, and promote electrolyte penetration into porous architectures. Cationic surfactants such as cetyltrimethylammonium bromide (CTAB) are especially effective in regulating nanostructure formation and tailoring electrochemical performance. CTAB not only directs nucleation and growth but also improves the interface between the active material and the electrolyte, thereby accelerating ion diffusion and redox kinetics [11,18,19]. While previous studies have used surfactants to enhance oxide materials, systematic studies on the effect of varying CTAB concentrations on the structure–performance relationship of NiFe_2_O_4_ electrodes remain exceptional. Understanding how the CTAB concentration affects the crystal phase, nanosheet morphology, and electrochemical response is critical for developing tailored electrode architectures with superior performance.

In this study, we report a CTAB-assisted hydrothermal route for synthesizing NiFe_2_O_4_ nanosheet electrodes and investigate the effect of the CTAB concentration (0.5%, 1%, and 1.5%) on the morphological evolution and electrochemical behavior. The structural, morphological, and redox characteristics of the resulting electrodes are thoroughly examined. The optimized NiFe-1 composition exhibits a highly interconnected nanosheet structure that enables enhanced electrolyte diffusion, charge storage, and cycling durability. The findings provide new insights into surfactant-assisted nanostructure engineering for high-performance ferrite-based supercapacitor systems.

## 2. Experimental Section

### Hydrothermal Synthesis of CTAB-Assisted NiFe Electrodes

**Figure 1:**
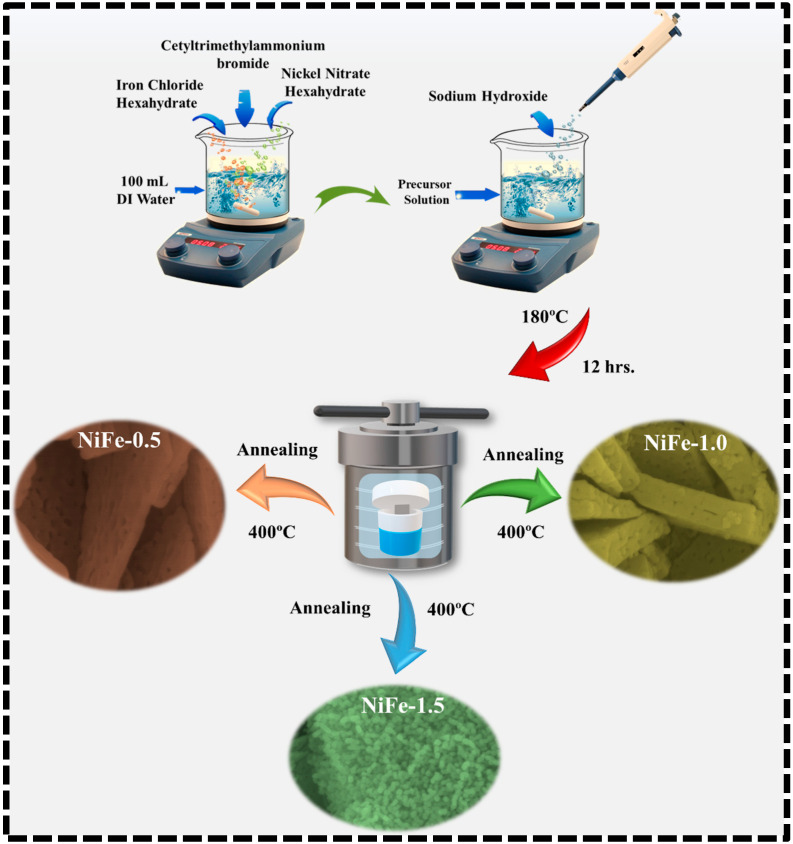
Depiction of hydrothermal synthesis of CTAB-assisted NiFe_2_O_4_ electrodes.

NiFe electrodes were synthesized on nickel foam via a hydrothermal method with varying concentrations of CTAB to enhance electrochemical properties. A precursor solution was prepared by dissolving 0.05 mol of Ni (NO_3_)_2_·6H_2_O (Sigma-Aldrich, St. Louis, MO, USA, 99.7%) and 0.1 mol of FeCl_3_·6H_2_O (Sigma-Aldrich, 99.99%, St. Louis, MO, USA) in 100 mL of deionized (DI) water under continuous stirring. To induce hydroxide precipitation and adjust the pH to 10, 0.06 mol of sodium hydroxide (NaOH (Sigma-Aldrich, 99.99%, St. Louis, MO, USA)) was gradually added under constant stirring. CTAB (Sigma-Aldrich, 99.9%, St. Louis, MO, USA) was introduced at concentrations of 0.5%, 1%, and 1.5%, followed by continuous stirring for 30 min to ensure uniform dispersion. Nickel foam (2 × 3 cm^2^) was sequentially cleaned via ultrasonication in 3 M HCl, ethanol, and DI water for 10 min each to remove surface oxides and contaminants. The cleaned foam was immersed in the precursor solution to facilitate uniform deposition. The mixture was then transferred into a Teflon-lined stainless-steel autoclave, sealed, and subjected to hydrothermal treatment at 180 °C for 12 h. After naturally cooling to room temperature, the coated nickel foam was removed, rinsed with DI water and ethanol, and dried at 80 °C overnight. To improve crystallinity and adhesion, the samples were annealed at 400 °C for 2 h in air. The synthesized electrodes, designated as NiFe-0.5, NiFe-1, and NiFe-1.5, corresponded to the CTAB concentrations of 0.5%, 1%, and 1.5%, respectively. Figure 1 shows the schematic illustration of the hydrothermal synthesis process for CTAB-assisted NiFe_2_O_4_ electrodes.

The CTAB concentrations of 0.5%, 1.0%, and 1.5% were chosen based on the literature and preliminary studies to represent conditions below, near, and above the critical micelle concentration (CMC) of CTAB (~1.0 wt%). This range enabled the systematic evaluation of micelle-assisted nucleation, anisotropic growth, and their impact on the morphology and electrochemical performance of NiFe_2_O_4_ electrodes.

## 3. Sample Characterization and Electrochemical Measurements

To evaluate the structural, morphological, and electrochemical characteristics of the synthesized CTAB-assisted NiFe_2_O_4_ electrodes, a comprehensive suite of analytical techniques was employed. An X-ray diffraction (XRD) (Bruker PAN Analytical, Almelo, The Netherlands) analysis was conducted using a PANalytical X’Pert PRO diffractometer equipped with Cu-Kα radiation (λ = 1.5406 Å), operating at 40 kV and 40 mA. Scans were recorded in the 2θ range of 10° to 80° at a step size of 0.02°/s. The resulting diffraction patterns were indexed using standard JCPDS files to confirm phase purity and crystallinity. Surface morphology and microstructural features were investigated using a field-emission scanning electron microscope (FE-SEM, Hitachi S-4800, Tokyo, Japan) operated at 5 kV under high vacuum (~10^−5^ Torr). Prior to imaging, samples were sputter-coated with a thin layer (~5 nm) of platinum to minimize surface charging. The FE-SEM analysis was coupled with energy-dispersive X-ray spectroscopy (EDS) (Hitachi S-4800, Tokyo, Japan) for elemental composition and mapping, using a silicon drift detector to ensure the accurate spatial distribution of Ni, Fe, and O within the nanostructure.

X-ray photoelectron spectroscopy (XPS) (K-alpha, Thermo Scientific, Cheshire, UK). was employed to study the chemical composition and oxidation states of the constituent elements. The measurements were carried out using a Thermo Kα + spectrometer with a monochromatic Al Kα source (hν = 1486.6 eV), operated at 12 kV and 6 mA under ultra-high vacuum conditions (~10^−9^ Torr). Survey and high-resolution scans were acquired using pass energies of 200 eV and 50 eV, respectively. All spectra were calibrated to the C 1s peak, and deconvolution was performed using a Shirley background with Voigt line shapes. Electrochemical measurements were performed using a Biologic WBCS3000 system (Seyssinet-Pariset, France) in a standard three-electrode configuration. The CTAB-assisted NiFe_2_O_4_ electrodes deposited on nickel foam served as the working electrode, while platinum wire and Ag/AgCl (3 M KCl) were used as the counter and reference electrodes, respectively. Aqueous 2 M KOH served as the electrolyte. Cyclic voltammetry (CV) tests were conducted over a potential range of −0.1 to 0.5 V at scan rates from 10 to 100 mV/s to evaluate redox behavior. Galvanostatic charge–discharge (GCD) measurements were carried out at current densities between 20 and 50 mA/cm^2^ to determine capacitance and rate capability. Electrochemical impedance spectroscopy (EIS) was performed in the frequency range of 100 kHz to 0.01 Hz with a 10 mV AC perturbation to assess the charge transfer resistance and equivalent series resistance [20,21].

## 4. Results and Discussions

### 4.1. XRD Elucidation

**Figure 2:**
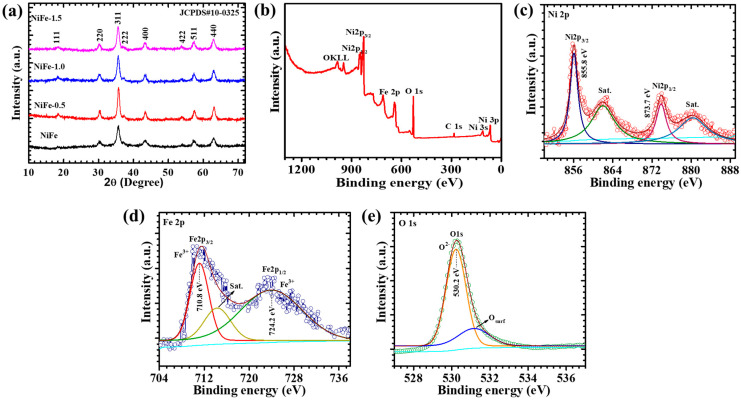
(**a**) XRD pattern; (**b**) XPS survey spectrum; high-resolution (**c**) Ni 2p, (**d**) Fe 2p, and (**e**) O 1s spectra of CTAB-assisted NiFe electrodes.

The structural integrity, crystallinity, and phase purity of the synthesized materials were thoroughly examined using an XRD analysis. Figure 2a presents the XRD spectra of pristine NiFe, NiFe-0.5, NiFe-1, and NiFe-1.5 electrodes, providing critical insights into their phase composition. The diffraction pattern of pristine NiFe exhibits distinct peaks at 18.2°, 30.1°, 35.8°, 43.3°, 53.7°, 57.5°, and 62.9°, which correspond to the (111), (220), (311), (400), (422), (511), and (440) crystal planes, respectively. These peak positions align well with the standard diffraction data from the JCPDS card No. 00-010-0325 confirming the successful formation of NiFe with the cubic phase. Additionally, the introduction of CTAB within the composite is indicated by prominent diffraction peaks observed in the NiFe-0.5, NiFe-1, and NiFe-1.5 electrodes. The NiFe-1 electrodes exhibit the diffraction peaks at 18.2°, 30.0°, 35.6°, 43.2°, 53.5°, 57.3°, and 62.8°, respectively. The absence of any additional diffraction peaks related to impurity confirms the formation of a single-phase material. The CTAB-assisted NiFe samples exhibit diffraction patterns similar to those of pure NiFe with a slight shift toward lower angles. A noticeable trend in the XRD patterns reveals that as the concentration of CTAB increases, the intensity of the corresponding diffraction peaks also intensifies, further validating the successful incorporation of CTAB into the composite matrix. The shift towards a lower angle indicates the presence of a more compact structural arrangement in the NiFe-0.5, NiFe-1, and NiFe-1.5 samples, likely due to the influence of CTAB in modulating the crystal lattice. These findings confirm the successful formation of the nanocomposite with well-defined crystallinity and phase purity [22].

Furthermore, the XRD-based microstructural analysis reveals that the crystallite size of the CTAB-assisted NiFe electrodes varies with the surfactant concentration, ranging from 10.5 to 13.2 nm, as summarized in Table 1. The average crystallite size is calculated using the Debye–Scherrer Equation (1) [23]:

(1) $$D=\frac{0.9\lambda }{\beta cos\theta }$$

where *D* is the crystallite size; *λ* is the wavelength of the X-ray radiation source; *β* is the full width at half maxima; *θ* is the Bragg angle (2*θ*). Further, the interplanar spacing calculated using Bragg’s relation (2) is as follows [23]:

(2) nλ=2dsinθ

The NiFe-1 sample exhibits the moderate crystallite size and lowest dislocation density (5.73 × 10^15^ lines/m^2^), indicating a reduced defect density. In contrast, the other samples show the highest dislocation density, suggesting more lattice imperfections. The lattice strain values also follow a decreasing trend with an increasing CTAB concentration, indicating improved crystallinity. The measured lattice spacing of 0.25 nm, corresponding to the (311) plane, and the lattice constant of 0.8292 nm confirm the formation of a well-defined cubic spinel structure. These microstructural parameters reflect the impact of CTAB on structural refinement, which directly influences the electrochemical performance by enhancing the charge transport and mechanical stability.

### 4.2. XPS Analysis

The XPS is employed to analyze the elemental composition, oxidation states, and electronic structure of the NiFe-1 electrode. The high-resolution XPS spectra are deconvoluted using Thermo Scientific Avantage software (Version No. 5.921). A Shirley background subtraction is applied, and the peaks are fitted using Voigt (Gaussian–Lorentzian mixed) functions with consistent full width at half maximum (FWHM) constraints. An XPS survey spectrum of the NiFe-1 electrode has been added in Figure 2b to provide comprehensive elemental identification. The survey spectrum confirms the presence of Ni, Fe, and O, validating the formation of the NiFe_2_O_4_ phase. The high-resolution Ni 2p spectrum (Figure 2c) displays two prominent peaks at 855.8 eV and 873.7 eV, corresponding to Ni 2p_3/2_ and Ni 2p_1/2_, respectively, which are characteristic of Ni^2+^ in the octahedral environment of a spinel NiFe_2_O_4_ lattice. Additionally, strong shake-up satellite peaks are observed at ~861.8 eV and ~880.3 eV, confirming the presence of high-spin Ni^2+^. The observed spin–orbit splitting of ~17.9 eV further supports this oxidation state assignment [13]. The Fe 2p spectrum (Figure 2d) exhibits binding energies at 710.8 eV (Fe 2p_3/2_) and 724.2 eV (Fe 2p_1/2_), along with distinct satellite peaks. The energy difference of ~13.4 eV between the Fe 2p doublet peaks is in good agreement with the Fe^3+^ oxidation state in transition metal oxides [14]. This confirms the successful incorporation of Fe^3+^ in the spinel structure. The O 1s spectrum (Figure 2e) is deconvoluted into two peaks: a dominant peak at 530.2 eV, attributed to lattice oxygen (O^2-^) in the metal–oxide matrix, and a secondary peak at 531.1 eV, ascribed to surface-adsorbed oxygen species such as hydroxyl groups (–OH), and defect-related oxygen. These oxygen species are associated with surface redox activity and play a key role in enhancing electrochemical performance by facilitating the ion exchange and active site accessibility. The presence of Ni^2+^ and Fe^3+^ in their respective valence states, along with oxygen vacancies or –OH functionalities, is consistent with the electrochemically active spinel NiFe_2_O_4_ phase. These findings validate the successful formation of the target composition with high redox activity and surface functionality [24,25].

### 4.3. Morphological and Elemental Composition

**Figure 3:**
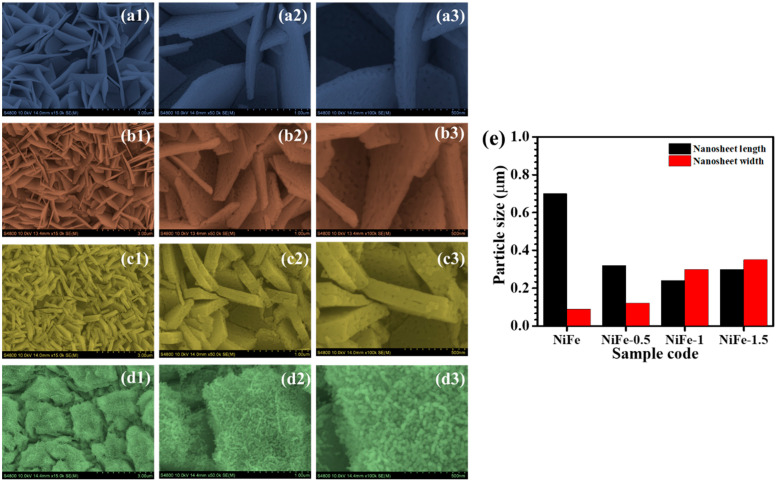
FE-SEM images of (**a1**–**a3**) bare NiFe, (**b1**–**b3**) NiFe-0.5, (**c1**–**c3**) NiFe-1, and (**d1**–**d3**) NiFe-1.5 samples at different magnifications; (**e**) nanosheets’ size distribution of the samples.

FESEM images in Figure 3 provide insight into the morphological evolution of NiFe electrodes with varying CTAB concentrations. The bare NiFe electrode (Figure 3a1–a3) exhibits a densely packed network of nanosheets forming a three-dimensional porous framework. These nanosheets appear smooth and interconnected, offering a high surface area, which is advantageous for electrochemical applications. However, the tightly packed arrangement may limit electrolyte penetration and ion transport, potentially restricting charge transfer efficiency [26]. Upon introducing 0.5% CTAB (Figure 3b1–b3), significant morphological changes are observed. The nanosheets become more dispersed, creating additional void spaces. This structural modification is attributed to the surfactant’s role in influencing nucleation and growth during synthesis. At higher magnifications, a slight increase in surface roughness is visible, indicating improved active site exposure. While this enhanced porosity can facilitate better electrolyte accessibility, the excessive separation of nanosheets may reduce electrical conductivity due to weakened interconnections [27]. With 1.0% CTAB (Figure 3c1–c3), the NiFe electrode exhibits a well-balanced structure with uniformly distributed and interconnected nanosheets. Compared to the NiFe-0.5 sample, this morphology shows an increased surface roughness and curling of nanosheets, which further enhance the active surface area. The optimized porosity ensures efficient electrolyte diffusion, reducing charge transfer resistance and improving ion transport. These structural advantages contribute to superior electrochemical performance by enabling fast redox reactions and enhanced charge storage capabilities. The well-maintained connectivity between nanosheets supports efficient electron conduction, making this composition highly favorable for electrochemical applications [28]. However, with 1.5% CTAB (Figure 3d1–d3), the nanostructure undergoes a drastic transformation. The nanosheets lose their distinct morphology, forming a more compact and irregular structure dominated by aggregated particles. High-magnification images reveal granular deposits covering the surface, suggesting that an excess of CTAB disrupts the controlled growth of nanosheets. This results in reduced porosity and restricted ion diffusion pathways, negatively impacting electrochemical performance by limiting the charge transfer and reducing the number of exposed active sites [29].

The morphological evolution of NiFe_2_O_4_ nanosheets with varying CTAB concentration (0.5%, 1%, and 1.5%) reveals a significant impact on both the length and width of the formed structures, as shown in Figure 3e. Initially, the pristine NiFe_2_O_4_ (without CTAB) displays elongated nanosheets with higher aspect ratios, indicative of anisotropic growth. Upon the addition of 0.5% CTAB, the nanosheet length is reduced by more than half, suggesting the onset of the surfactant-mediated control over growth kinetics. This effect becomes more pronounced at 1% CTAB, where nanosheets become significantly shorter and wider, reflecting a more isotropic morphology. At the highest concentration of 1.5% CTAB, a slight increase in length is observed, but the width continues to grow, leading to thicker and more aggregated structures formed by nanoparticles. This development suggests that beyond a critical micelle concentration, CTAB not only acts as a growth moderator but also promotes lateral agglomeration and the broadening of sheets.

**Figure 4:**
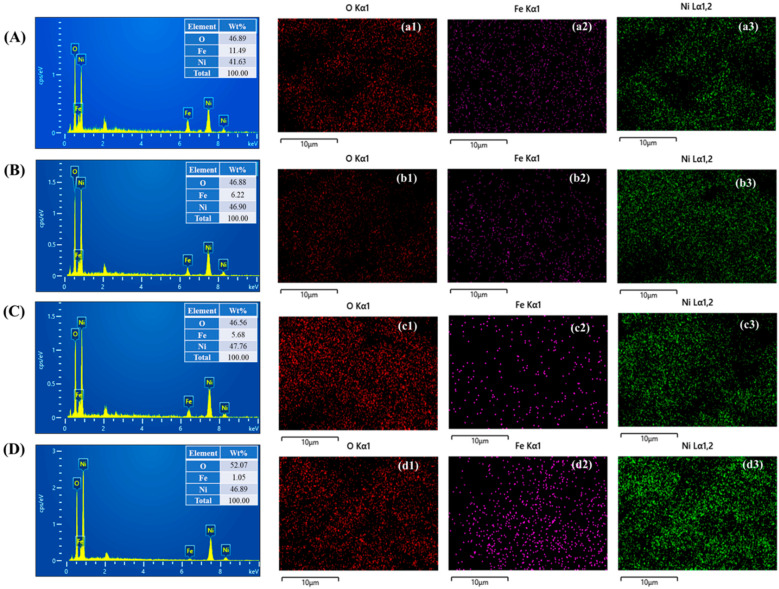
EDS spectra and elemental mapping of (**A,a1**–**a3**) bare NiFe, (**B,b1**–**b3**) NiFe-0.5, (**C,c1**–**c3**) NiFe-1, and (**D,d1**–**d3**) NiFe-1.5 samples.

An EDS analysis is conducted to determine the elemental composition of surfactant-assisted NiFe electrodes, including bare NiFe, NiFe-0.5, N iFe-1, and NiFe-1.5. The corresponding EDS spectra, presented in Figure 4A–C, confirm the presence of iron (Fe), nickel (Ni), and oxygen (O) in all samples, verifying the successful formation of NiFe_2_O_4_ on the nickel foam substrate. The insets in Figure 4A–C display the weight percentage (wt%) distribution of these elements. Additionally, the spatial distribution of elements within the hydrothermally synthesized electrodes is assessed through EDS elemental mapping. As illustrated in Figure 4a1–d3 for bare NiFe, NiFe-0.5, NiFe-1, and NiFe-1.5, the mapping images reveal a uniform dispersion of Fe, Ni, and O across all surfactant-assisted samples. This even distribution highlights the homogeneity and consistent structural integrity of the synthesized electrodes, further emphasizing their suitability for electrochemical applications [30].

## 5. Electrochemical Analysis

**Figure 5:**
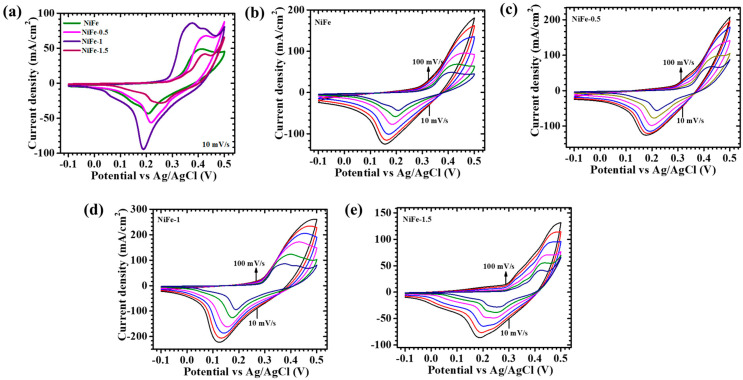
Cyclic voltammetry of (**a**) all NiFe electrodes at a scan rate of 10 mV/s, in a potential window −0.1 to 0.5 V; cyclic voltammetry of (**b**) bare NiFe, (**c**) NiFe-0.5, (**d**) NiFe-1, and (**e**) NiFe-1.5 samples at different scan rates (10–100 mV/s).

The electrochemical properties of NiFe electrodes synthesized with and without varying CTAB concentrations (0.5%, 1%, and 1.5%) are systematically investigated using cyclic voltammetry (CV), galvanostatic charge–discharge (GCD), and electrochemical impedance spectroscopy (EIS) in a three-electrode configuration with a 2M KOH electrolyte. Figure 5a displays the CV profiles recorded within a potential range of −0.1 V to 0.5 V at a scan rate of 10 mV/s, demonstrating distinct redox peaks across all samples. The presence of these peaks confirms the faradaic nature of charge storage, indicative of reversible redox reactions inherent to NiFe. The CV response is further analyzed across a range of scan rates (10–100 mV/s) for NiFe, NiFe-0.5, NiFe-1, and NiFe-1.5, as depicted in Figure 5b–e. A consistent increase in current density is observed with an increase in scan rate while maintaining the characteristic shape of the CV curves, signifying stable electrochemical behavior and efficient charge storage. The largely symmetric nature of the CV curves suggests rapid ion transport and an enhanced charge storage mechanism [31]. Notably, the NiFe-1 electrode exhibits a more pronounced redox peak intensity and a larger enclosed CV area compared to the other samples, indicating a higher charge storage capacity. This enhancement is attributed to the optimized electrode structure facilitated by the controlled CTAB concentration, which significantly improves the electroactive surface area and ion diffusion pathways. The morphological influence of CTAB on the NiFe electrode’s performance is evident, as the NiFe-1 sample displays a well-balanced structure with uniformly distributed and interconnected nanosheets’ features. This architecture provides a uniform network conducive to enhanced ion accessibility and charge transfer kinetics [32]. In contrast, NiFe-0.5 and NiFe-1.5 exhibit lower current densities, likely due to unfavorable morphological characteristics. The NiFe-0.5 electrode demonstrates a less-developed structure, limiting the number of active sites for redox reactions, whereas NiFe-1.5 exhibits increased compact and aggregated morphological characteristics, restricting electrolyte penetration and ion transport. On the other hand, the NiFe sample shows poor electrochemical performance due to its suboptimal nanostructure. The electrochemical redox process governing the charge storage in NiFe electrodes can be represented by the following reaction (3) [15]:

(3) $$Ni{Fe}_{2}O_{4}+H_{2}O+{OH}^{-}⇌NiOOH+FeOOH+e^{-}$$

**Figure 6:**
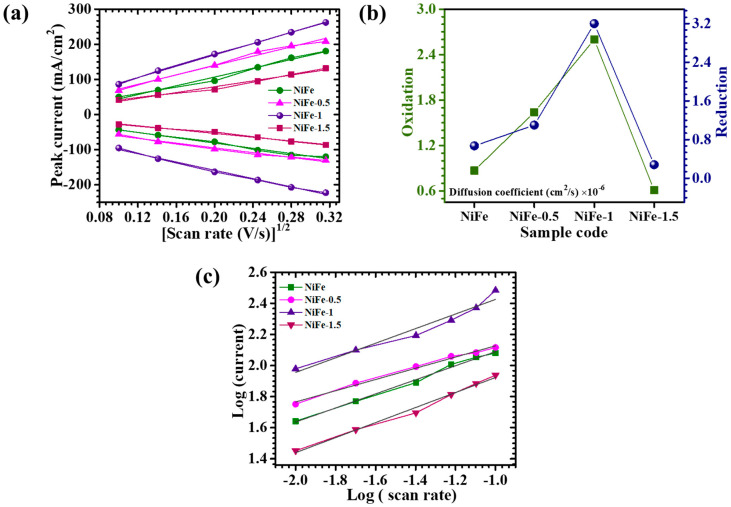
(**a**) Plot of peak current vs. (scan rate)^1/2^; (**b**) graphical representation for diffusion coefficients obtained for each NiFe_2_O_4_ electrode; (**c**) plot of *log*(*i*) against the *log*(*ϑ*).

The CV curves acquired at varying scan rates (10–100 mV/s) are further analyzed to elucidate the underlying redox mechanisms. Figure 6a presents the correlation between the peak current (*i_p_*) and the square root of the scan rate (*v*^1/2^) for each electrode, revealing a linear trend. This direct proportionality suggests that the charge storage process is diffusion-controlled, with ion transport dynamics playing a significant role in electrochemical performance. To gain deeper insights into charge transfer kinetics, the diffusion coefficients of NiFe, NiFe-0.5, NiFe-1, and NiFe-1.5 electrodes are estimated using the *i_p_* and *v*^1/2^ data. The diffusion coefficient (D) at a scan rate of 10 mV/s is calculated based on the Randles–Sevcik Equation (4) [33]:

(4) $$D=\frac{i_{p}}{2.69\times 10^{5}\times n^{3/2}\times A\times C\times v^{1/2}}$$

where *i_p_* denotes the peak current density, *v*^1/2^ represents the square root of the scan rate, *n* corresponds to the number of electrons involved in the redox process, *A* is the effective surface area of the working electrode (cm^2^), *C* is the electrolyte ion concentration, and *D* is the diffusion coefficient. Table 2 summarizes the diffusion coefficients obtained for each NiFe electrode, while Figure 6b provides a graphical representation for comparative evaluation. Among the investigated samples, NiFe-1 exhibits the highest diffusion coefficient for both oxidation and reduction reactions, suggesting enhanced ion mobility and superior charge transport properties. The variation in diffusion coefficients among the electrodes can be attributed to differences in the electrode nanostructure, influenced by the CTAB surfactant concentration. The NiFe-1 electrode, synthesized with an optimized CTAB concentration, displays a well-balanced structure with a uniformly distributed and interconnected nanosheet network, which facilitates improved electrolyte penetration and accelerated ion diffusion [34]. Conversely, NiFe, NiFe-0.5, and NiFe-1.5 exhibit relatively lower diffusion coefficients, likely due to their compact and agglomerated morphologies, which restrict efficient electrolyte interactions with electroactive sites. The enhanced ion transport kinetics observed in NiFe-1 underscore its potential for high-performance energy storage applications, emphasizing the critical role of CTAB in tailoring electrode architecture to optimize electrochemical properties.

Dunn’s method is a well-established approach for evaluating the b-value, a crucial parameter that defines the dominant charge storage mechanism in electrode materials. This technique involves plotting *log*(*i*) against the *log*(*v*), as illustrated in Figure 6c, where the slope of the resultant linear fit determines the b-value. In this study, Dunn’s method is employed to analyze the charge storage characteristics of NiFe electrodes using the mathematical relationships given in Equations (5) and (6) [35,36]:

(5) $$i={av}^{b}$$

(6) $$\log\left(i\right)=\log\left(a\right)+b\;log(v)$$

Here, ‘*a*’ and ‘*b*’ are constants derived from the intercept and slope of the *log*(*i*) vs. *log*(*v*) plot. The b-value serves as an indicator of the charge storage mechanism, with values close to ~1 suggesting capacitive-dominated behavior, and values around ~0.5 indicating a diffusion-controlled process. The calculated b-values for NiFe, NiFe-0.5, NiFe-1, and NiFe-1.5 electrodes are found to be in the range of 0.4 to 0.7 (Table 2), implying that the electrochemical charge storage in these electrodes is predominantly governed by a diffusion-controlled process. This behavior suggests that charge storage occurs primarily through ion intercalation, electrolyte diffusion, and redox activity at the electrode–electrolyte interface [37].

**Figure 7:**
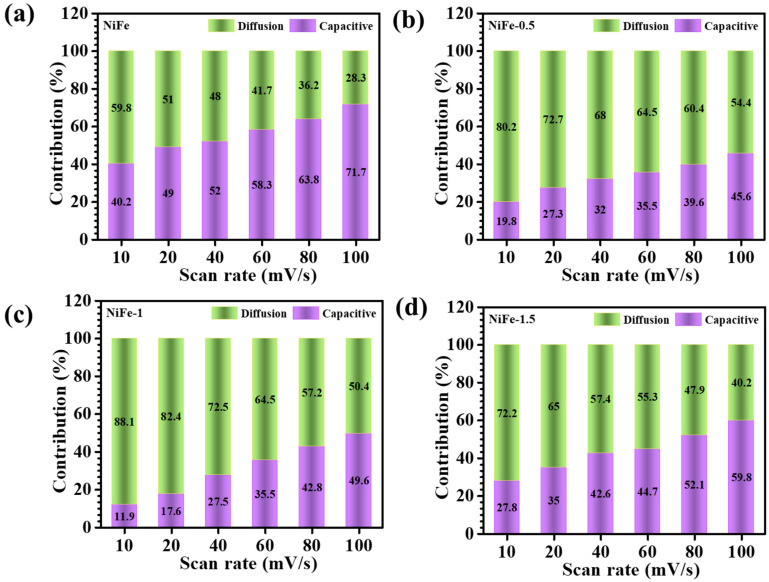
Capacitive and diffusion-controlled processes at different scan rates of (**a**) bare NiFe, (**b**) NiFe-0.5, (**c**) NiFe-1, and (**d**) NiFe-1.5 samples.

To further distinguish the relative contributions of diffusion-controlled and capacitive-controlled currents, the total current response is deconvoluted using Equation (7) [38]:

(7) $$i\left(V\right)=k_{1}v{+k_{2}v}^{1/2}$$

where *i*(*V*) represents the current at a given potential *V*, while *k*_1_ and *k*_2_ correspond to the capacitive and diffusion-controlled contributions, respectively. The values of *k*_1_ and *k*_2_ are extracted from the linear fitting of *i*(*V*)*/*$v^{1/2}$ vs. $v^{1/2}$, enabling the quantification of both charge storage components. The diffusion-controlled contributions at the 10 mV/s scan rate for NiFe, NiFe-0.5, NiFe-1, and NiFe-1.5 are determined to be 59.8%, 80.2%, 88.1%, and 72.2%, respectively. Notably, NiFe-1 exhibits the highest diffusion-controlled contribution, which can be attributed to its well-balanced structure with a uniformly distributed and interconnected nanosheets’ structure. The well-interconnected uniform network and the presence of multiple oxidation states in NiFe facilitate a greater number of electroactive sites, leading to enhanced faradaic redox activity and efficient ion transport. Additionally, the effect of varying scan rates on the capacitive and diffusion-controlled contributions is assessed for all electrodes, as presented in Figure 7a–d. The results reveal an increase in capacitive-controlled charge storage at higher scan rates. This phenomenon occurs because, at elevated scan rates, ion diffusion into the bulk electrode material becomes kinetically constrained, thereby promoting surface-dominated charge storage. Consequently, capacitive behavior becomes more prominent under fast-charging conditions, whereas diffusion-controlled mechanisms remain dominant at lower scan rates [13]. These observations highlight the influence of CTAB-assisted structural modifications on the electrochemical performance of NiFe electrodes, demonstrating the tunability of charge storage mechanisms through morphological engineering.

**Figure 8:**
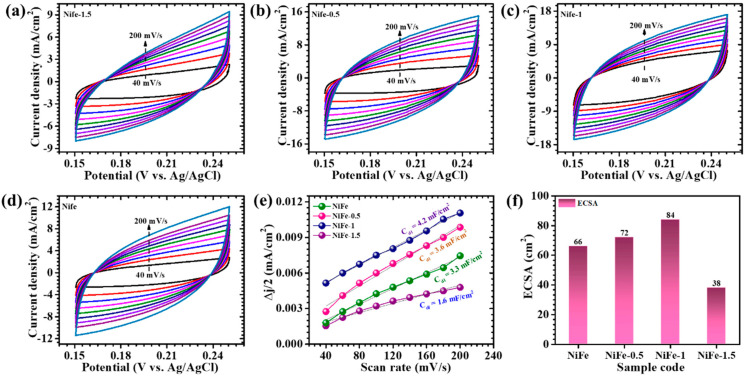
(**a**–**d**) CV measurements at various scan rates within a non-faradaic region for all electrodes; (**e**) plot of resulting current variations at different scan rates for assessments of double-layer capacitance (C_dl_); and (**f**) the computed ECSA values for the NiFe, NiFe-0.5, NiFe-1, and NiFe-1.5 electrodes.

To evaluate the electrochemically active surface area (ECSA) of CTAB-assisted NiFe electrodes, CV measurements are conducted at various scan rates (40–200 mV/s) within the non-faradaic region, as depicted in Figure 8a–d. This approach allows for the precise determination of the electrode’s capacitive behavior, avoiding faradaic processes that could interfere with the measurements. The resulting current variations at different scan rates are plotted to assess the double-layer capacitance (*C_dl_*) of the electrodes, illustrated in Figure 8e. The *ECSA* is estimated using the following Equation (8) [39],

(8) $$ECSA=\frac{C_{dl}}{C_{s}}$$

where *C_dl_* is the double-layer capacitance, and *C_s_* represents the specific capacitance of the sample (0.04 mF cm^−2^). This provides a reliable way of quantifying the active surface that contributes to electrochemical reactions, which is crucial for understanding the material’s performance in energy storage applications. The computed ECSA values for the NiFe, NiFe-0.5, NiFe-1, and NiFe-1.5 are about 66 cm^2^, 72 cm^2^, 84 cm^2^, and 38 cm^2^, respectively, as shown in Figure 8f. Notably, the highest ECSA value observed for the NiFe-1 sample indicates an optimally porous surface structure, facilitating enhanced electrolyte accessibility and improved ion transport. This directly correlates into a superior electrochemical performance, highlighting the critical role of a specific surface area in supercapacitor applications. Specifically, a higher electrochemically active surface area enhances the interaction between the electrode surface and electrolyte ions, thereby significantly improving the charge storage capability and overall electrode efficiency.

**Figure 9:**
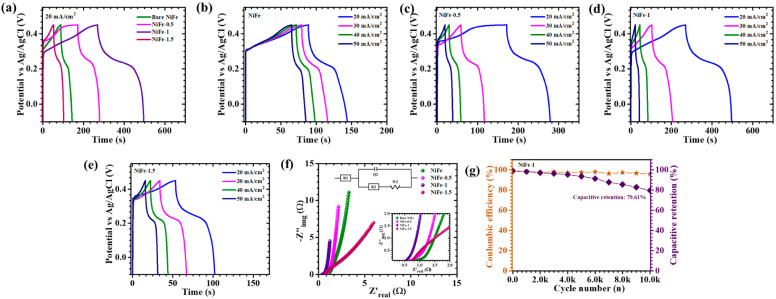
(**a**) GCD curves of bare NiFe, NiFe-0.5, NiFe-1, NiFe-1.5 sample electrodes at 20 mA/cm^2^ current density; GCD plot of (**b**) bare NiFe, (**c**) NiFe-0.5, (**d**) NiFe-1, and (**e**) NiFe-1.5 electrodes at different current densities; (**f**) EIS analysis of NiFe electrodes (inset: EIS fitted circuit); and (**g**) cyclic stability over 10,000 GCD cycles of NiFe-1 sample.

The electrochemical storage characteristics of NiFe electrodes are analyzed through GCD testing, performed at a current density of 20 mA/cm^2^ within a voltage range of −0.1 to 0.45 V, as shown in Figure 9a. The resulting discharge curves display noticeable deviations from linearity, indicative of redox-driven charge storage rather than purely electrostatic capacitance. Such behavior confirms the pseudocapacitive nature of the electrodes, where Faradaic reactions contribute significantly to charge retention. Further, the rate capability is assessed by varying the current density from 20 to 50 mA/cm^2^ (Figure 9b–e) for with and without CTAB-assisted NiFe electrodes. The derived electrochemical parameters using Equations (9)–(11) [40], areal capacitance (*C_A_*), energy density (*ED*), and power density (*PD*), are summarized in Table 3.

(9) $$C_{A}\left({F/cm}^{2}\right)=\frac{I_{d}\times T_{d}}{A\times dV}$$

(10) $$ED\left(Wh{F/cm}^{2}\right)=\frac{C_{A\times }{dV}^{2}}{2\times 3600}$$

(11) $$PD\left(W{/cm}^{2}\right)=\frac{ED\times 3600}{T_{d}}$$

Notably, the NiFe-1 electrode exhibits the highest areal capacitance of 8.21 F/cm^2^ at 20 mA/cm^2^, an energy density of 0.3453 mWh/cm^2^, and a power density of 5.5 mW/cm^2^. This retention highlights its remarkable ability to sustain performance under increasing charge–discharge rates. The observed electrochemical enhancement in NiFe-1 is closely linked to the morphological modifications induced by the controlled addition of CTAB during synthesis. CTAB plays a critical role in directing the growth of a well-structured, interconnected nanosheet network, facilitating efficient ion diffusion and electron transport. This optimized architecture enhances redox-active site accessibility, ensuring superior charge storage efficiency [15]. The variation in performance across different CTAB concentrations underscores the importance of surfactant-assisted structuring in fine-tuning electrode properties for high-performance energy storage applications.

The EIS is conducted at a fixed potential of 10 mV to analyze the charge transport dynamics at the electrode–electrolyte interface, as depicted in Figure 9f. This technique provides essential insights into the resistive and capacitive characteristics of the system. The Nyquist plot, illustrating the relationship between the imaginary (−Z″) and real (Z′) components of impedance, is employed to assess the equivalent series resistance (ESR, R1). The ESR, which represents the intrinsic resistance of the electrode material, is determined from the high-frequency intercept on the *x*-axis [41]. To gain deeper insights into the interfacial charge transport and ion diffusion behavior of the CTAB-assisted NiFe electrodes, the Nyquist plot is fitted using an equivalent circuit model (inset of Figure 9f). The extracted fitting parameters are summarized in Table S2. Among the samples, the NiFe-1 electrode exhibits the lowest charge transfer resistance (R_2_ = 0.1337 Ω) and minimal solution resistance (R_1_ = 0.3789 Ω), indicating superior conductivity and interfacial charge transfer. This reduction in resistance can be attributed to the optimized uniform architecture formed through the controlled incorporation of CTAB during synthesis. The well-defined uniform nanosheets significantly enhance ion mobility and minimize charge transfer resistance, leading to improved electrochemical performance. In contrast, other samples (e.g., NiFe, NiFe-0.5, and NiFe-1.5) show higher R_2_ and s_2_values, suggesting less efficient ion transport, likely due to less favorable morphologies or partial agglomeration.

For practical energy storage applications, long-term electrochemical stability is a critical parameter in determining the reliability of electrode materials. The cycling endurance of the NiFe-1 electrode is systematically evaluated through 10,000 consecutive GCD cycles at a high-current density of 70 mA/cm^2^. The corresponding trends in capacitance retention and coulombic efficiency over the cycling period are presented in Figure 9g. After enduring 10,000 cycles, the NiFe-1 electrode demonstrates remarkable stability, sustaining 79.61% of its initial capacitance while maintaining an impressive coulombic efficiency of 95.76%. The electrode exhibits exceptional structural resilience, with minimal degradation observed in the first 4000 cycles, highlighting its robust electrochemical integrity. The superior cycling performance can be a result from the well-engineered uniform morphology induced by CTAB-assisted synthesis, which enhances electrolyte penetration and facilitates efficient charge transport. This rigid structure mitigates mechanical stress and prevents material disintegration, ensuring long-term stability even under high-current conditions. These results establish NiFe-1 as a promising candidate for next-generation electrochemical energy storage applications, combining high durability with efficient charge storage capabilities.

To assess the structural stability of the NiFe-1 electrode, a post-cycling FESEM analysis is carried out following extended electrochemical cycling. As illustrated in the FESEM images (Figure S1a,b), the NiFe-1 sample largely retains its nanosheet-like morphology even after prolonged charge–discharge operation. The nanosheets remain well-defined, with no substantial structural collapse, delamination, or severe particle agglomeration observed. While minor surface roughening and edge thinning are evident—likely resulting from repeated ion insertion/extraction processes—a majority of the nanosheets remain structurally intact, demonstrating only minimal degradation. These morphological observations highlight the excellent mechanical integrity and structural resilience of the CTAB-assisted NiFe_2_O_4_ electrode, which correlate well with its sustained electrochemical performance and high cycle stability.

In addition to their high performance in electrochemical energy storage, the optimized CTAB-assisted NiFe-1 nanosheets, featuring high porosity, a large electrochemically active surface area, and redox-active sites, also exhibit strong potential for multifunctional applications. Such structural and electronic characteristics are advantageous for gas sensing, particularly for the detection of volatile organic compounds (VOCs) and for environmental monitoring, as highlighted in the recent literature [42]. Moreover, the low equivalent series resistance and outstanding cycling stability demonstrated by this electrode suggest its viability for integration into low-power electronic systems. Notably, its stable and efficient energy output could support emerging ultra-low-power technologies, such as single-photon avalanche diode-based sensors, enabling the development of compact, self-powered sensor platforms for use in environmental, biomedical, and portable electronic applications. This highlights the broader applicability and interdisciplinary relevance of the NiFe_2_O_4_ material system developed in this study.

## 6. Electrochemical Performance of Asymmetric Supercapacitor Device

**Figure 10:**
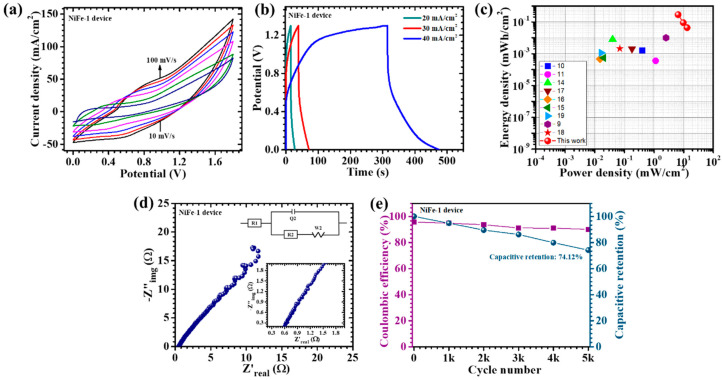
(**a**) CV tests performed on the NiFe-1//AC device recorded at a scan rate of 10–100 mV/s across a potential range of 0 to 1.8 V; (**b**) GCD measurements at different current densities for NiFe-1//AC device; (**c**) Ragone plot; (**d**) EIS measurement of the device (inset: EIS fitted circuit); (**e**) cyclic stability of 5000 GCD cycles of NiFe-1//AC device.

To explore the practical application of NiFe electrodes beyond the conventional three-electrode setup, an APSD was constructed and thoroughly evaluated. The device configuration utilized NiFe-1 as the positive electrode and activated carbon (AC) as the negative electrode, both supported on nickel foam. The separator was composed of filter paper saturated with a 2M KOH electrolyte, and the entire device was sealed to protect it from environmental exposure. The electrochemical performance of the APSD was investigated through CV, GCD, and EIS. As shown in Figure 10a, the CV curves at varying scan rates confirmed a stable operational window from 0 to 1.8 V, which is conducive to significant energy storage. The CV tests, conducted at scan rates ranging from 10 to 100 mV/s, demonstrated a consistent increase in the current response, indicative of efficient charge transfer and excellent reversibility within the device. This behavior highlights the synergistic interaction between NiFe-1 and AC, which contributes to the enhanced overall electrochemical performance of the device. The GCD measurements, conducted at different current densities (Figure 10b), exhibited non-linear discharge profiles, signifying a dominant pseudocapacitive mechanism and an efficient charge–discharge process with minimal energy loss. At a current density of 20 mA/cm^2^, the device achieved a remarkable areal capacitance of 1.215 F/cm^2^, an energy density of 0.285 mWh/cm^2^, and a power density of 6.5 mW/cm^2^, emphasizing the device’s ability to store and release significant energy per unit area. The energy–power density relationship of the fabricated asymmetric supercapacitor device based on the NiFe-1 electrode is illustrated in the Ragone plot (Figure 10c). The red symbols represent the performance metrics achieved in this work, while the other colored symbols correspond to previously reported transition metal oxide-based supercapacitor systems from the literature. Notably, the device in this study demonstrated a superior combination of energy and power densities, with values reaching up to 0.2853 mWh/cm^2^ at a power density of 6.5 mW/cm^2^. Even at higher power densities (13 mW/cm^2^), the energy density remained well-retained, highlighting the device’s excellent rate capability and fast charge–discharge characteristics (Table 4). Compared to other reports, the NiFe-1//AC device exhibited an outstanding electrochemical performance, outperforming many previously published systems in both energy and power metrics. This superior performance can be attributed to the nanosheet-like morphology, enhanced porosity, and improved ion transport pathways induced by the optimized CTAB-assisted synthesis. Overall, the Ragone plot validates the practical applicability of the NiFe-1 electrode in high-performance energy storage devices. The EIS analysis (Figure 10d) further confirmed the excellent electrochemical properties of the APSD, revealing a low equivalent series resistance (R_1_) of 0.48 Ω, which indicated high electrical conductivity and efficient ion transport at the electrode–electrolyte interface. The reduced ESR supported the device’s outstanding rate capability and energy storage efficiency. The device demonstrated a promising performance with a low R_2_ = 0.1077 Ω, though a relatively higher Warburg impedance (s_2_ = 46.97 Ω/s^1/2^) (Table S2) reflected the added complexity of the complete asymmetric pouch-type configuration. Furthermore, the long-term cycling stability is a critical performance metric for supercapacitors. The APSD exhibited impressive durability, retaining 74.12% of its initial capacitance after 5000 consecutive GCD cycles at a current density of 70 mA/cm^2^ (Figure 10e). Additionally, the device maintained a high coulombic efficiency of 90.1%, demonstrating excellent charge retention and minimal energy loss over extended cycling. This exceptional stability can be attributed to the well-balanced structure with the uniformly distributed and interconnected nanosheet structure of the NiFe-1 electrode, which enhanced electrolyte accessibility, minimized particle aggregation, and mitigated material degradation during repeated cycling. The incorporation of an optimized CTAB concentration during the electrode synthesis was instrumental in achieving this stable, well-defined morphology, ensuring the long-term electrochemical performance of the device [43].

In addition to its electrochemical performance, the practical aspects of the device were also considered. The synthesis route is cost-effective and scalable, relying on inexpensive precursors and minimal CTAB content (1%), making it suitable for large-scale production using batch or continuous hydrothermal systems. Although current cycling tests reflect the long-term operational durability under accelerated conditions, further evaluation under real-world variables such as temperature, humidity, and mechanical stress will be undertaken in future work to fully assess the environmental robustness of the device. These aspects highlight the realistic potential of the APSD for practical energy storage applications.

## 7. Conclusions

This study highlights the critical role of CTAB-assisted morphology engineering in enhancing the electrochemical properties of NiFe_2_O_4_-based electrodes. Among the investigated samples, the NiFe-1 electrode, synthesized with 1% CTAB, exhibited an optimized nanosheet architecture characterized by uniform distribution, high porosity, and an enhanced electrochemically active surface area. This structural optimization led to an impressive areal capacitance of 8.21 F/cm^2^, along with a high energy density of 0.3453 mWh/cm^2^ and a power density of 5.5 mW/cm^2^ at 20 mA/cm^2^. A kinetic analysis demonstrated a dominant diffusion-controlled charge storage mechanism, with 88.1% contribution at 10 mV/s, further validating the suitability of this material for high-performance pseudocapacitive applications. Notably, the electrode maintained 79.61% of its initial capacitance over 10,000 charge–discharge cycles, underscoring its long-term cycling durability. The EIS revealed a low charge transfer resistance of 0.37 Ω, confirming efficient ion/electron transport. The practical applicability of the NiFe-1 electrode was further demonstrated through the successful fabrication of an asymmetric pouch-type supercapacitor device (NiFe-1//AC), which operated within a wide window and retained 74.12% of its capacitance after 5000 cycles. Beyond energy storage, the morphological and electrochemical attributes of the CTAB-assisted NiFe_2_O_4_ nanosheets suggest a promising multifunctional potential for integration into next-generation energy storage devices. Overall, this work not only offers a scalable and tunable route for surfactant-guided nanostructure design but also contributes a high-performance electrode material for advanced energy storage technologies.

## Figures and Tables

**Table 1:** Crystallite size, lattice strain, and dislocation density of CTAB-assisted NiFe_2_O_4_ electrodes calculated from XRD data.

Sample Code	Crystallite Size (D, nm)	Lattice Strain	Dislocation Density (line/m^2^) × 10^15^
**NiFe**	10.5	0.01123	9.07
**NiFe-0.5**	13.2	0.00889	5.83
**NiFe-1**	11.25	0.01052	5.73
**NiFe-1.5**	11.3	0.01042	7.9

**Table 2:** Estimated diffusion coefficient and b-values of bare NiFe, NiFe-0.5, NiFe-1, and NiFe-1.5 electrodes.

Sample Code	Diffusion Coefficient (cm^2^/s) × 10^−6^		b-Value
	Oxidation	Reduction	
**NiFe**	0.87	0.67	0.77
**NiFe-0.5**	1.64	1.1	0.6
**NiFe-1**	2.6	3.2	0.42
**NiFe-1.5**	0.61	0.28	0.66

**Table 3:** Evaluation of calculated areal capacitance, specific capacity, energy density, and power density values of bare NiFe, NiFe-0.5, NiFe-1, and NiFe-1.5 electrodes.

Sample Code	I (mA)	CA (F/cm^2^)	C (mAh/cm^2^)	ED (mWh/cm^2^)	PD (mW/cm^2^)
**NiFe**	20	1.96	0.5455	0.0825	5.5
	30	1.8	0.5253	0.0749	8.25
	40	1.78	0.4949	0.0665	11
	50	1.72	0.4798	0.0535	13.75
**NiFe-0.5**	20	2.03	0.5657	0.0856	5.5
	30	1.96	0.5455	0.0825	8.25
	40	1.58	0.4394	0.0749	11
	50	1.45	0.4040	0.0611	13.75
**NiFe-1**	20	8.21	2.2828	0.3453	5.5
	30	4.1	1.1364	0.1719	8.25
	40	3.2	0.8889	0.1344	11
	50	2.09	0.5303	0.0802	13.75
**NiFe-1.5**	20	1.16	0.3232	0.0489	5.5
	30	1.14	0.3182	0.0481	8.25
	40	1.06	0.3030	0.0458	11
	50	0.91	0.2525	0.0382	13.75

**Table 4:** Calculated energy storage parameters of NiFe-1//AC asymmetric pouch-type supercapacitor device.

Sample Code	I (mA)	CA (F/cm^2^)	C (mAh/cm^2^)	ED (mWh/cm^2^)	PD (mW/cm^2^)
**NiFe-1 device**	20	1.215	0.3376	0.2853	6.5
	30	0.380	0.1058	0.0894	9.75
	40	0.184	0.0513	0.0433	13

## Data Availability

The data presented in this study are available on request from the corresponding author. The data are not publicly available due to restrictions..

## Supplementary Materials

The following supporting information can be downloaded at: https://www.mdpi.com/article/10.3390/ma18091987/s1, Figure S1: (a,b): FESEM images of NiFe-1 electrode after long-term cycling stability; Table S1: Energy storage parameters comparison of the current study with existing literature; Table S2: Fitted EIS parameters of CTAB-assisted NiFe_2_O_4_ electrodes and the NiFe-1 based asymmetric device.
