# Supplementary material for: Cationic Surfactant-Driven Evolution of NiFe2O4 Nanosheets for High-Performance Asymmetric Supercapacitors
Source: Materials (Basel). 2025 Apr 27;18(9):1987. doi: 10.3390/ma18091987 (PMC12072712; doi:10.3390/ma18091987)
Supplement: Supplementary file 1 [file materials-18-01987-s001.zip › materials-3596383-supplementary.pdf]

Supplementary Information

**Material**	**Areal capacitance (F/cm^2)**	**Energy density (mWh/cm^2)**	**Power density (mW/cm^2)**	**Cycle stability**	**Reference**
$NiFe_2O_4$	2.271 (in H_2SO_4)<br>1.845 (in KOH)	4.14	-	91%(3000 cycles)	11
$NiFe_2O_4$	1.414 1	1.6776 1	10.588	-	12
$NiFe_2O_4$	0.472	0.94	-	98%(7000 cycles)	13
HMTA-$NiFe_2O_4$	1.1242	0.6982	-	80.3% (1500 cycles)	21
$NiFe_2O_4$	0.4818	1.015	0.28	130%(2000 cycles)	15
$NiFe_2O_4$ QDs@Ni-MOF	1.7046	0.65	1.596	87.3% (5000 cycles)	34
**NiFe-1.0**	**8.21**	**0.3453**	**5.5**	**79.61%(10000 cycles)**	**This work**

Table S1: Energy storage parameters comparison of the current study with existing literature.

Sample	R1 (Ω)	Q2	R2 (Ω)	s2 ($\Omega/s^{1/2}$)
**NiFe**	0.6835	0.01127	0.5517	2.871
**NiFe-0.5**	0.5151	0.143	0.463	3.401
**NiFe-1**	0.3789	0.05655	0.1337	1.122
**NiFe-1.5**	0.7252	0.142	0.4681	3.383
**NiFe-1 Device**	0.4814	0.005872	0.1077	46.97

Table S2: Fitted EIS parameters of CTAB-assisted $NiFe_2O_4$ electrodes and the NiFe-1 based asymmetric device.

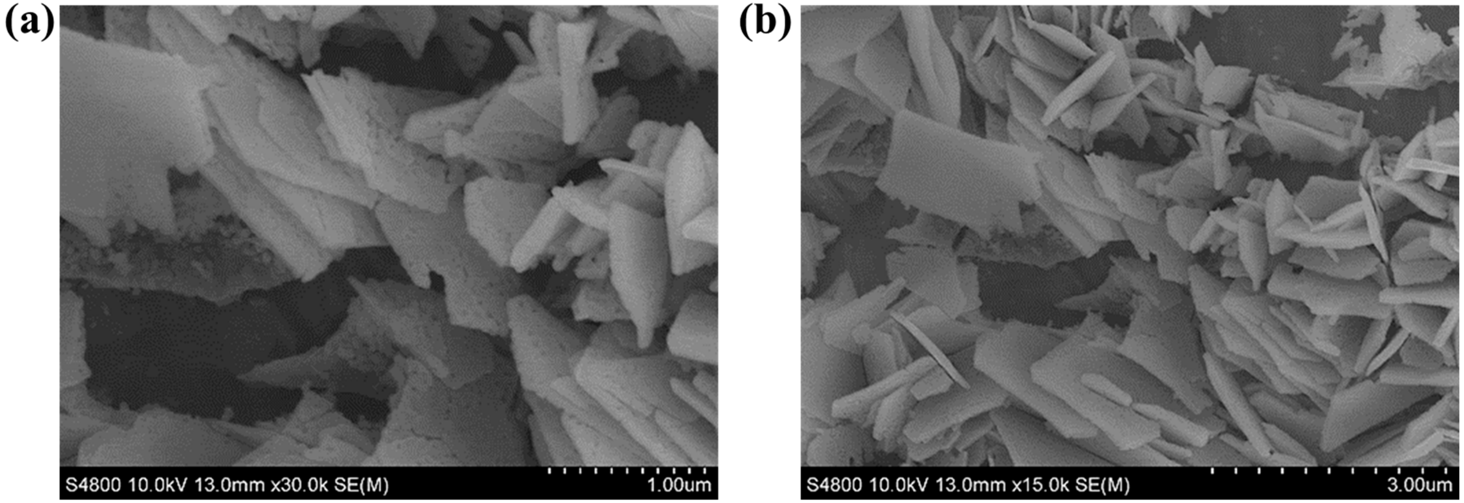

Figure S1 (a, b): FESEM images of NiFe-1 electrode after long-term cycling stability.
